# A Telerehabilitation Approach to Enhance Quality of Life Through Exercise Among Adults With Paraplegia: Study Protocol

**DOI:** 10.2196/resprot.8047

**Published:** 2017-10-19

**Authors:** Shane Norman Sweet, Meredith Rocchi, Kelly Arbour-Nicitopoulos, Dahlia Kairy, Brigitte Fillion

**Affiliations:** ^1^ Center for Interdisciplinary Research in Rehabilitation of Greater Montreal (CRIR) Department of Kinesiology and Physical Education McGill University Montreal, QC Canada; ^2^ Faculty of Kinesiology and Physical Education University of Toronto Toronto, ON Canada; ^3^ Center for Interdisciplinary Research in Rehabilitation of Greater Montreal (CRIR) École de réadaptation Université de Montréal Montreal, QC Canada; ^4^ Center for Interdisciplinary Research in Rehabilitation of Greater Montreal (CRIR) Centre de réadaptation Lucie-Bruneau Centre intégré universitaire de santé et de services sociaux (CIUSSS) du Centre-Sud-de-l’Île-de-Montréal Montreal, QC Canada

**Keywords:** telehealth, spinal cord injuries, exercise, motivation, telerehabilitation

## Abstract

**Background:**

Despite compelling evidence linking physical activity and quality of life among adults with spinal cord injury (SCI), exercise participation rates are extremely low in this population. Unfortunately, a lack of behavioral exercise interventions, in particular theory-based randomized controlled trials (RCT), exists within the SCI literature. A pilot RCT is needed to first examine the feasibility to conduct such interventions and determine the appropriate effect size to inform future full-scale interventions.

**Objective:**

The overall goal of this pilot RCT is to test an 8-week innovative, video-based telerehabilitation intervention based on self-determination theory and aimed at enhancing the basic psychological needs, motivation, exercise participation, and quality of life‒related outcomes of adults with paraplegia. The objectives are to (1) determine if individuals in the intervention group have greater increases in their basic psychological needs and autonomous motivation and a decrease in controlled motivation compared to the control group, (2) determine whether the intervention group reports greater increases in exercise participation and quality of life‒related variables (eg, life satisfaction, participation in daily/social activities, depressive symptoms) compared to the control group, and (3) examine if adults with paraplegia who received the intervention report improved scores on psychosocial predictors of exercise (eg, action planning) and well-being (eg, positive affect) compared to the control group. We also aimed to examine the implementation characteristics of the intervention (eg, satisfaction with the technology, counselor’s ability to foster the psychological needs).

**Methods:**

Adults with paraplegia (N=24) living in the community will be recruited. All participants will be invited to complete assessments of their psychological needs, motivation, exercise, and quality of life‒related variables at three time points (baseline, 6, and 10 weeks). Following the baseline assessment, participants will be randomly assigned to the intervention or control group. Participants in the intervention group will participate in 8 weekly, 1-hour video-based telerehabilitation sessions with a trained physical activity counselor, while participants in the control group will be asked to continue with their regular routine.

**Results:**

We expect higher ratings of the basic psychological needs and autonomous motivation and lower scores for controlled motivation for the intervention group compared to the control group (Objective 1). We also expect that our video-based intervention will have moderate effects on exercise participation, as well as small-to-moderate positive effects on the quality of life‒related variables (Objective 2). Finally, we expect the intervention to have a small positive effect on psychosocial predictors of physical activity and well-being (Objective 3).

**Conclusions:**

We anticipate that the results will show that the intervention is appropriate for adults with paraplegia and feasible to test in a full-scale RCT.

**Trial Registration:**

ClinicalTrials.gov NCT02833935; https://clinicaltrials.gov/ct2/show/NCT02833935 (Archived by WebCite at http://www.webcitation.org/6u8U9x2yt)

## Introduction

Quality of life (QOL) and participation in daily/social activities have become important research topics in spinal cord injury (SCI) [1]. One health behavior that has shown potential to positively influence these psychosocial outcomes is exercise. Exercise consists of any physical activities that are structured and planned and have the objective of improving or maintaining physical fitness [2]. Systematic reviews have demonstrated the important relationship between exercise and QOL among adults with SCI [3]. These reviews provide empirical support for exercise as a viable intervention strategy to enhance QOL in the SCI population.

Despite the compelling evidence linking exercise and QOL among adults with SCI, participation rates are extremely low in this population. One large epidemiological study (N=695) showed that 50% (n=348) of adults with SCI participated in 0 minutes of exercise [4] and that this inactive group was mostly represented by male participants over age 33 who had 11 or more years since injury. Exercise participation among adults with SCI remains bleak when we look at the percentage of individuals who are meeting the SCI-specific physical activity (ie, exercise activity) recommendations that were published in 2011 [5]. According to these guidelines, adults with SCI should participate in at least two 20-minute bouts of aerobic activity per week and two strength training activities per week, both at a moderate-to-vigorous intensity [5]. Using this benchmark, only 12% (n=9) of a sample of 73 adults with SCI met these guidelines [6]. Given the low participation rates, a clear need exists among the SCI population to find strategies to promote physical activity.

### Exercise Interventions Among Adults With Spinal Cord Injury

Unfortunately, few behavioral exercise interventions have been tested among adults with SCI [7]. The majority of the six SCI behavioral exercise intervention studies outlined by Nery et al were delivered in person [7]. Given that transportation is an important barrier among adults with SCI who may want to participate in trials [8] and that transportation-related costs are an obstacle to exercise participation as a whole [9], the reach of in-person interventions is limited to individuals who live in proximity to the research sites or who can easily pay to commute to the sites.

Recent technological advances within the rehabilitation context [10] allow for other alternative and viable options for delivering cost-effective and equitable exercise interventions to community-dwelling adults with SCI. One such example is telerehabilitation, which consists of delivering rehabilitative and health-related services from a distance with the use of telecommunications [11]. A systematic review found that telerehabilitation was as successful as traditional, in-person rehabilitation across various settings and populations [10]. Of importance to exercise promotion, telerehabilitation has the advantage of delivering the intervention in the person’s natural environment and is an effective tool in the self-management of chronic conditions, which includes increasing exercise participation [12]. Thus, telerehabilitation has the potential to be a viable alternative for community dwelling adults with SCI.

Telerehabilitation has also been shown to be effective at managing mental health outcomes among adults with SCI [13-15]. For instance, Dorstyn et al [13] conducted a systematic review of telerehabilitation and found, across seven studies (N=272), that telerehabilitation was effective at improving short-term psychological (eg, depression) and functional outcomes and was time- and cost-effective. Furthermore, physicians, nurses, psychologists, and physical/occupational therapists have effectively applied telerehabilitation to consult on a wide range of outcomes such as pressure ulcers, depression, and functional motor skills [14-17].

In terms of exercise participation, a telerehabilitation phone-based intervention was conducted among adults with SCI, which consisted of a 6-month national telephone-based exercise counseling service (called Get in Motion) for Canadian adults with SCI [18]. This service was derived from two previous randomized controlled trials (RCT) [19,20]. Results from this telephone counseling service demonstrated that individuals’ intentions to be active remained high throughout the 6 months. The percentage of adults with SCI who participated in regular exercise (defined as ≥30 minutes of moderate-to-vigorous intensity activity on ≥3 days/week) increased from 35% (n=16) at baseline to 52% (n=24) at 6 months. This study provided initial evidence that telephone-based telerehabilitation interventions could be an effective strategy for increasing exercise participation among community-dwelling adults with SCI. Moving to video-based telerehabilitation will allow the counselor to capture nonverbal messages, demonstrate exercises, and see the person’s natural environment in order to better adapt the counseling (a need that has been previously mentioned by clients using the Get In Motion service [21]). To our knowledge, no study has conducted a video-based telerehabilitation intervention to promote exercise participation among adults with SCI. Because no such intervention exists, we propose to develop and evaluate the effectiveness of this type of telerehabilitation intervention, which will also fill the current gap in SCI-specific exercise intervention research [7].

### Self-Determination Theory

**Figure 1:**
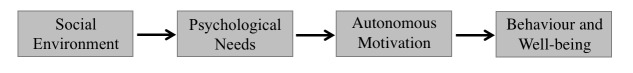
Overview of self-determination theory.

Experts have strongly recommended that exercise interventions be grounded in psychological theory to enhance their effectiveness [22,23]. Self-determination theory (SDT [24,25]; see Figure 1) is a motivational theory based on a humanistic perspective that acknowledges every human being has an innate tendency towards growth, integration, and well-being. According to SDT, satisfaction of the three psychological needs is essential for promoting motivation and well-being [26]. The three basic psychological needs of autonomy (ie, volition in one’s actions), competence (ie, belief in one’s actions), and relatedness (ie, sense of belongingness). In SDT, motivation is conceptualized as either autonomous, where people engage in behaviors because they want to, or controlled, where they engage in behaviors because they perceive they have to [27,28].

As elaborated in SDT, the social environment is central to facilitating the satisfaction of the three basic psychological needs. Exercise counselors who adopt an interaction style that is consistent with SDT principles (ie, a need supportive style) would create such an environment by fostering and helping satisfy the basic psychological needs of autonomy, competence, and relatedness. A key element to support the need for autonomy is to elicit and acknowledge individuals’ perspectives and life aspirations. Support for competence is intended to enhance participants’ perceptions that they have the ability to attain their behavior change goals. An empathic, nonjudgmental approach aimed at understanding participants’ viewpoint would facilitate the need for relatedness.

Previous research supports the tenet that satisfaction of the psychological needs predicts greater autonomous motivation which, in turn, predicts greater exercise behavior [29,30]. SDT-based interventions have also shown success with positively influencing psychological needs fulfillment, autonomous motivation, exercise behavior, and QOL [31-33]. Despite the success of SDT-based physical activity interventions, no such intervention has been applied among adults with SCI.

To our knowledge, only a few published studies have examined SDT-based concepts in adults with a physical disability and they used primarily correlational designs [34,35]. A recently published study demonstrated that autonomous motivation had both direct and indirect relationships with exercise activity among adults with SCI [6]. Specifically, the results showed that autonomous motivation was positively related to the likelihood of meeting the physical activity guidelines for adults with SCI. Taken together, these results suggest that the principles of SDT are applicable to adults with a disability, including SCI.

### Proposed Research

Given the compelling evidence linking exercise participation and QOL, the low rates of exercise participation among adults with SCI, and a lack of exercise participation interventions, in particular SDT-based, telerehabilitation RCTs, a pilot RCT is needed. The advantage of a pilot RCT is that it allows us to determine the feasibility and acceptability of the intervention, design, and procedures prior to running a full-scale RCT. Furthermore, the effect sizes derived from this pilot RCT can help estimate an appropriate effect size for the full-scale RCT [36].

**Figure 2:**
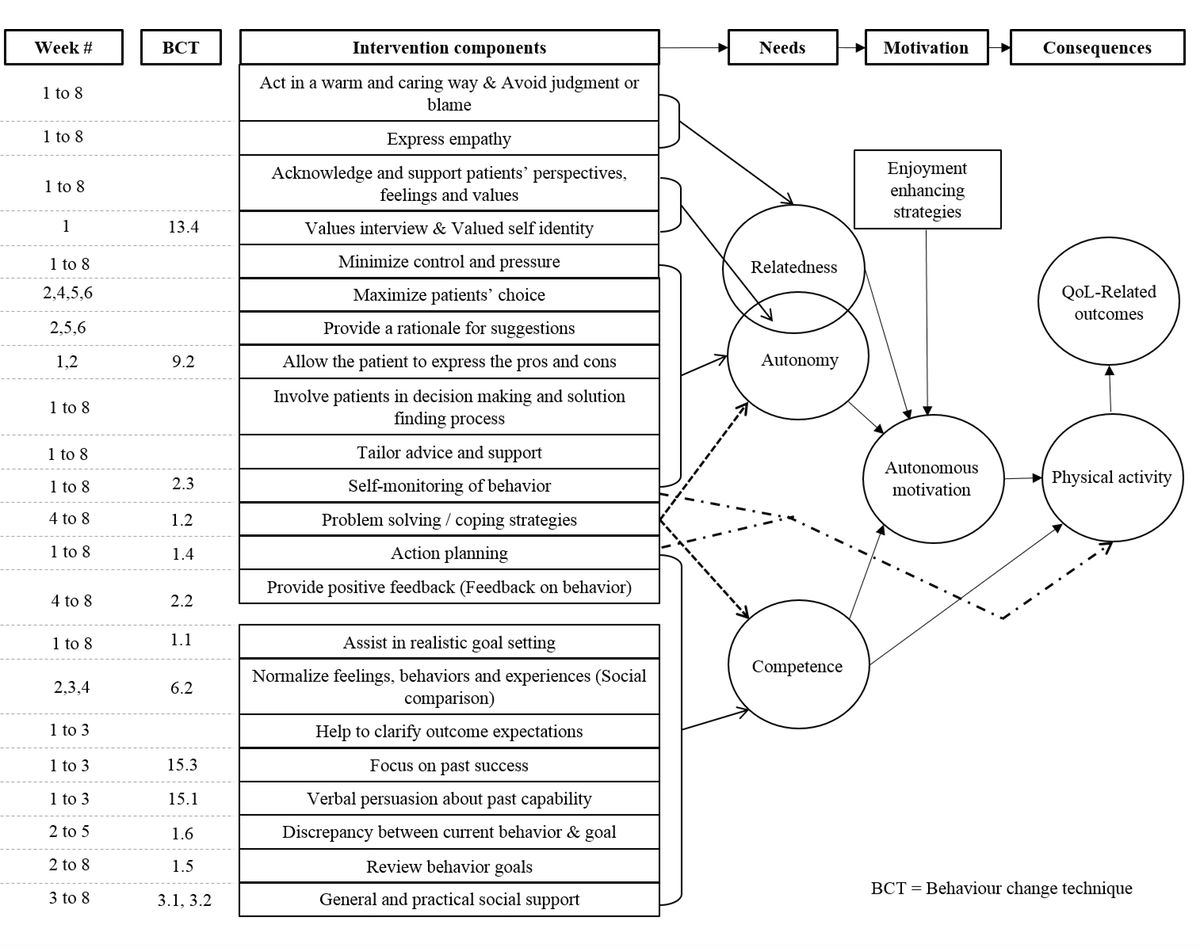
Conceptual model with intervention components of the proposed pilot RCT (with permission from Canadian Science Publishing to use a modified version of the figure appearing in Fortier et al [39]).

The development of effective interventions should also be grounded in sound theory given the conceptual and methodological advantages of theory-based interventions outlined earlier. Although most interventions claim to be guided by theory, the majority does not link the intervention components to the theoretical constructs being tested [37,38]. To avoid this common flaw, we have implemented the Theory Coding Scheme [38] into the design of this pilot RCT, as recommended by Gourlan et al [37]. By following this coding scheme, we are ensuring that our intervention is theory-based. The proposed intervention is also novel for the SCI field as, to date, no exercise interventions have used SDT as the guiding framework. We will therefore be the first to extend the generalizability of SDT to adults with SCI, which is an important and often disregarded step of theory testing. Figure 2 illustrates the conceptual and process model that guides this proposed intervention [39].

### Specific Aims

The overall goal of this pilot RCT is to test an 8-week innovative, SDT- and video-based telerehabilitation intervention aimed to enhance the basic psychological needs, motivation, exercise participation, and QOL-related outcomes of adults with paraplegia. The primary purpose of this pilot RCT is to determine if individuals in the intervention group have greater increases in their basic psychological needs and autonomous motivation and a decrease in controlled motivation, compared to the control group. We expect moderate effects on these SDT variables based on the results from previous SDT exercise interventions among the general population [40].

The secondary purpose of this study is to determine if the intervention group reports greater increases in exercise behavior compared to the control group. Additionally, we will examine if adults with paraplegia who received the intervention report improved scores on QOL-related variables (eg, life satisfaction, participation in daily/social activities, depressive symptoms) compared to the control group. Given that moderate effects were found for exercise behavior in the previously mentioned Get In Motion service and theory-based exercise interventions [18,37], we hypothesize, for Objective 2, that our video-based intervention will have moderate effects on exercise participation. We anticipate small-to-moderate positive effects of the intervention group compared to the control group on the QOL-related outcomes of life satisfaction, participation in daily/social activities, and depressive symptoms given the results of a leisure activity (including exercise participation) intervention [41] and cross-sectional research [3,42] in adults with SCI. We are expecting smaller effects for these variables given the relatively short duration of the proposed pilot RCT.

Our tertiary purpose is to investigate group differences in additional and common psychosocial predictors of exercise participation (eg, action planning), as well as indicators of well-being (eg, positive affect), to obtain a broader perspective of the impact of the intervention. Similarly, we expect moderate differences between the intervention and control groups on these variables.

Finally, we are conducting an implementation evaluation to determine satisfaction with the technology and extent to which the counselor delivered the intervention as intended. No hypotheses are derived for this evaluation given its description nature.

## Methods

### Participants

We will recruit 24 adults with paraplegia from three rehabilitation centers, one adapted fitness center, provincial SCI organizations, and through social media. We will randomize 12 participants to the intervention group and 12 to the control group. Based on previous interventions [18], we expect a 15% dropout rate resulting in 20 individuals who will complete the proposed pilot RCT.

Eligible participants will be adults with paraplegia who (1) have sustained their injury at least 1 year prior, (2) are over the age of 18 years, (3) have access to a computer, and (4) speak and understand English or French. Eligible participants will either have the intention to become physically active in the next 2 months (ie, not amotivated as per SDT) or have been minimally active (<2 times a week [5]) in the past 2 months. Participants will be excluded if they are receiving in-patient rehabilitation services, been diagnosed with memory impairments, severe communication difficulties and/or severe visual impairments, do not require a mobility device (eg, wheelchair, cane), or have answered yes to one of the questions on the Physical Activity Readiness Questionnaire (PAR-Q) and the SCI questions on the PAR-QX. Participants who have answered yes to the PAR-Q could be eligible if they provide a note from their physician stating that it is safe for them to participate in exercise activities. We have elected to focus on adults with paraplegia who use a mobility device because we want to pilot test the intervention in a more homogeneous sample than if we included both adults with tetraplegia and paraplegia.

### Procedures

#### Registration

This trial is funded by the Craig H. Neilsen Foundation through their psychosocial research grants funding program. As a first step, this trial was officially registered at ClinicalTrials.gov (#NCT02833935). This is an online official protocol registration and results system.

#### Study Protocol

Interested participants will meet over the phone with a research assistant to assess their eligibility for the study, provide their informed consent, and confirm that they meet the requirements for installing the Remote Education, Augmented Communication, Training, and Supervision (REACTS) software (Innovative Imaging Technologies), which is the video-based telerehabilitation software and that they know how to use the software. REACTS is an interactive audio-video software that enables secure live communication and interaction between two or more individuals over an encrypted network. REACTS also allows for multifeed streaming where multiple webcams can be connected and for multimedia sharing. All sessions can also be videorecorded if the participant accepts the recording. In addition to using the video-audio and secure features, the exercise counselor also enables the multimedia sharing platform where both she and the participants can edit a shared document (eg, action plans). Both the participant and the exercise counselor use a Windows-based computer meeting the software requirements.

Next, participants will be invited to complete the baseline questionnaire either verbally over the phone (with an emailed or mailed copy of the questionnaire to follow along with) or electronically using an online survey platform (SurveyGizmo). If the questionnaire package is mailed, a 1-day courier service will be used to minimize the delay. Once the baseline questionnaire is completed, the research assistant will randomly assign participants to the intervention or control group by opening a blinded, prelabeled (1-24), randomly ordered envelope to assign participants to one of two groups. The randomization is also stratified by gender (16 men, 8 women) in attempt to have a representative sample by gender [43]. The envelopes will be prepared by another member of the research team not directly involved in the data collection. The random allocation will be determined by a randomization tool (randomizer.org). Participants will be informed of their group assignment and told that another research assistant, blinded to their group allocation, will contact them to complete the same questionnaire at two other time points (6 weeks and 10 weeks from baseline). These follow-up data collection time points (6 weeks and 10 weeks) are set to represent the mid and end of the 8-week intervention, which will start 2 weeks after baseline. Participants will receive up to Can $100 for completing the study. Specifically, they will receive $30, $35, and $35 if they complete the baseline, 6-week, and 10-week questionnaires, respectively.

#### Intervention Group

For participants assigned to the intervention group, the research assistant will schedule the participants’ first intervention session with the exercise counselor 2 weeks after their baseline assessment. The 2-week delay will allow us to send intervention-related materials (eg, webcams) and train participants on the REACTS software.

#### Intervention Format

The exercise intervention will be delivered by a kinesiologist trained in behavioral counseling and the adapted exercise in a Web-based face-to-face format through the REACTS interactive audio-video software. Intervention participants will receive 1 weekly exercise session for 8 weeks. An 8-week intervention was chosen (1) because the 8-week mark was associated with the greatest increases in Get in Motion clients’ exercise participation behavior [18] and represented the time point with the highest number of dropouts [21], and (2) to ensure feasibility for completion of the pilot RCT. The exercise counselor will receive behavior change skills training to ensure she is capable of fostering the psychological needs within SDT and apply behavior change techniques. The counselor will also participate in a motivational interviewing workshop to fine-tune her counseling skills. The use of motivational counseling intervention methods do not have any detrimental effects on exercise participation levels as motivationally focused interventions have been shown to be as effective as structured exercise interventions [44]. Across all of the eight intervention sessions, the counselor will create a social environment that is congruent with SDT.

#### Intervention Components

The exercise counselor will individually tailor her approach to each participant by understanding and taking into consideration the participant’s past and current exercise experiences (including a discussion of any prescribed exercise programs after completing an outpatient rehabilitation program), motives to be physically active (eg, improve functional ability, enhance participation in daily/social activities), salient concerns and barriers regarding exercise participation, and their physical home environment. Throughout the intervention, the exercise counselor and participants will co-construct and collaboratively adapt the participants’ exercise goals. SDT is at the core of this proposed intervention and a Theory Coding Scheme [38] will be used to evaluate the extent to which the theory is used, applied, and tested within the intervention. In line with the Theory Coding Scheme, we have linked each of our intervention components to either the three basic psychological needs, autonomous motivation, and/or exercise participation as illustrated in Figure 2. The specific weeks that each intervention component is planned to be implemented is shown in the first column of Figure 2 (Week #). Although the intervention components have been attributed to specific counseling sessions/weeks, the timing of the intervention components may differ between participants. The intervention is not standardized because, to be in line with SDT, the intervention will be tailored to the participants. As such, the participants’ interests and goals will be at the forefront of each session, and some components may be used earlier or later than outlined in Figure 2. Thus, the exercise counselor will be trained to use the model as a guide rather than a set protocol.

#### Control Group

Participants assigned to the control group will be asked to continue with their regular routine for the next 2 months. We recognize that a compensatory rivalry bias [45] may occur as participants in the control group may seek out their own exercise program, which may then reduce the intervention’s effect. To help minimize this bias, the research assistant will remind control participants, after they complete the baseline questionnaire, of the importance of keeping their regular routine for the following 10 weeks. The control group will also be offered an exercise counseling session following the completion of the 10-week data collection time point.

### Measures

Participants will be invited to complete each of the primary, secondary, and tertiary outcome measures at all three time points (baseline, 6, and 10 weeks). Participants in the intervention group will also complete the relevant measures under implementation outcomes.

#### Primary Outcomes

##### Basic Psychological Needs

The Psychological Need Satisfaction in Exercise Scale will be used to assess the satisfaction of the psychological needs for exercise [46]. On a 6-point Likert scale ranging from 1 (false) to 6 (true), participants will respond to 18 items reflecting how they might feel when physically active. A mean will be calculated for autonomy (6 items; “I feel free to exercise in my own way”), competence (6 items; “I feel that I am able to complete exercises that are personally challenging”), and relatedness (6 items; “I feel close to my exercise companions who appreciate how difficult exercise can be”). A higher value on each need will indicate greater satisfaction for that need [46].

##### Autonomous and Controlled Motivation

Two scales will be used to assess participants’ motivation. The Behavioral Regulation Exercise Questionnaire-3 will be used to assess participants’ motivation for why they usually engage in exercise activities [47,48]. Participants will respond to 23 items, on a 5-point Likert scale ranging from 0 (not true for me) to 4 (very true for me), covering the types of motivational regulations on the self-determination continuum. The mean score of participants’ autonomous and controlled motivation will be calculated. This questionnaire has been shown to be reliable and valid [47,48]. Additionally, the Treatment Self-Regulation for Exercise Scale [49] will be used to assess changes in autonomous and controlled motivation for reasons why one would engage in exercise activities. Participants will respond to 15 items, using a 7-point Likert scale ranging from 1 (not at all true) to 7 (very true). Again, a mean score of participants’ autonomous and controlled motivation will be calculated.

#### Secondary Outcomes

##### Moderate-to-Vigorous Exercise

This will be assessed using the self-report 7-day Leisure-Time Physical Activity Questionnaire for Adults with SCI [50]. This scale was validated among adults with SCI. Participants will be asked to indicate the frequency (days) and duration (in minutes) they engage in mild (ie, activities that require little effort), moderate (ie, activities that require some physical effort that make you feel like you are working somewhat hard, but you feel like you can keep going for a long time), and vigorous intensity (ie, activities that require a lot of physical effort that make you feel like you are working really hard, almost at your maximum, and you can do the activity only for a short period of time before getting tired) activities over the last 7 days. Weekly minutes of total activity (mild, moderate, and vigorous) and of moderate and vigorous activity will be summed.

##### Life Satisfaction

The Life Satisfaction Questionnaire-11 [51] is a standardized and validated QOL measure that asks 11 questions about satisfaction in various areas of life, including life in general, vocation, financial situation, leisure, social/friends/family, sexual life, family life, and physical and mental health (1=very dissatisfying to 6=very satisfying). The mean score of the 11 items will be calculated for this measure. We have previously used this scale among adults with SCI [52].

##### Participation in Daily/Social Activities

The Patient-Perceived Participation in Daily Activities Questionnaire will be used to assess participation as it was developed for the SCI population [53]. Participants will be presented with a list of 26 activities. For each activity, they are asked, “Do you participate in this activity?” Response options are “Yes, as much as I want” (4); “Yes, but less than I want” (3); “No, but I would like to” (2); and “No, but I don’t want to” (1). An overall participation in daily/social activities score will be calculated as well as six subscale scores reflecting broad categories of participation (the reliability indices from a previous study are also noted for each subscales [54]):

- autonomous participation – indoors (7 items, eg, performing bladder care; Cronbach alpha=.88)
- autonomous participation – outdoors (6 items, eg, carrying out productive activities that are unpaid, like volunteering; alpha=.74)
- family roles (4 items, eg, carrying out family responsibilities; alpha=.66)
- health (2 items, eg, maintaining your physical health; *r*=.25)
- social relationships (4 items, eg, maintaining relationships with others; alpha=.62)
- work-education (3 items, eg, participating in activities that prepare you to start working in a paid job; alpha=.71)

##### Depressive Symptoms

The 9-item Patient Health Questionnaire (PHQ-9) [55] will be used to assess self-reported depressive symptoms. Participants will be asked, “Over the past 4 weeks, how often have you been bothered by any of the following problems?” and rate each symptom (eg, little interest or pleasure in doing things, poor appetite or overeating) on a 4-point scale (0=not at all; 3=nearly every day). The PHQ-9 has been suggested as a valid and reliable tool for the SCI population [56] and demonstrated to have strong psychometric properties in people with SCI [42,57]. A mean score of the items will be computed.

#### Tertiary Outcomes

##### Psychosocial Predictors of Exercise

Participants will complete the Social Cognitive Predictors of Leisure Time Physical Activity among adults with SCI [58]. This measure consists of a battery of short questionnaires assessing additional psychosocial predictors of leisure time exercise activity such as self-efficacy, intentions, and action planning among adults with SCI.

##### Well-Being

Participants will also complete the Positive and Negative Affect Schedule Questionnaire [59], a 10-item mood scale. This is a measure of affective experience capturing broader aspects of well-being/QOL. Finally, participants will complete the Meaning Questionnaire [60], which is a questionnaire designed to measure meaningful life experiences. This 5-item scale asks participants to rate their responses from 1 (absolutely untrue) to 7 (absolutely true) and demonstrates strong reliability (Cronbach alpha=.88).

#### Implementation Outcomes

Participants’ perceptions of the exercise counselor will be assessed with a modified Health Care Climate Questionnaire [61]. This questionnaire assesses participants’ perceived need support from their exercise counselor. Participants will respond to six items (eg, “My exercise counselor listened to how I would like to do things regarding my exercise”) on a 7-point Likert scale ranging from strongly disagree (1) to strongly agree (7). High alpha levels (.88-.95) have been demonstrated in previous studies [62,63]. They will also respond to an adapted Short Feedback Questionnaire to obtain information about the participants’ experience in using technology, in this case the REACTS telerehabilitation system. Participants will rate eight questions addressing the sense of presence, enjoyment, control, ease of use and discomfort using a 5-point Likert scale ranging from not at all (1) to a lot (5). This measure has been shown to have good internal consistency and moderate concurrent validity [64].

To ensure that our intervention was delivered as intended, we have included implementation and feasibility evaluation procedures. First, we will ask the exercise counselor to fill out a checklist for each session with each participant. The checklist will include questions regarding the logistics of the session (eg, time, components of the REACTS software used), and a list of the intervention components. We will also assess the exercise counselors’ satisfaction with the REACTS technology with the validated Technical Quality Subjective Appreciation questionnaire. The questionnaire is divided in two sections: 1) five items relate to the technical quality of the sessions (audio, video); and (2) three questions relate to the counseling objectives, relationship with the patient, and overall satisfaction. The exercise counselor will rate each item on a score from 0 (“Bad”) to 3 (“Good”). The mean score of each section will be calculated. Second, we will videorecord all eight counseling sessions of consenting participants. These sessions will be evaluated by the research assistant and principal investigator by using the aforementioned checklist to determine the consistency of the reporting by the exercise counsellor. In addition, we will evaluate the extent to which the counselor was able to provide a social environment that supported the psychological needs within SDT. By conducting this implementation and feasibility evaluation, we will gain valuable information as to the implementation of the intervention, which will help inform the intervention for the subsequent larger trial.

## Results

### Anticipated Timelines

Prior to starting the RCT, the exercise counselor will be trained in SDT-based counseling, motivational interviewing, and adapted exercise activity. The exercise counselor will also pilot the intervention with an experienced counselor and 2 inactive participants in order to troubleshoot any potential issues. The exercise counselor and research assistants will be also trained on the REACTS software system.

At the time of submission of this protocol, data collection was ongoing with 10 participants who had completed the study (5 intervention, 5 control) and 6 who were enrolled in the study (3 intervention, 3 control). At the time of publication, all 24 participants have been randomized.

### Planned Data Analyses

Data will be screened for statistical outliers and assumptions for each statistical test will be examined [65]. Participant attrition is a common problem in trial studies and case-wise deletion can bias results [66]. As such, missing data will be examined to determine the pattern of missing data and imputed with multiple imputations methods that are appropriate for small samples [67]. Further, we will collect reasons for missing data/attrition. These approaches will help inform the expected attrition rate, reasons why people did not continue with the trial, and required sample size to conduct a subsequent larger RCT. Once data are cleaned and imputed, demographic and injury-related covariates will be examined for each outcome through a correlation matrix. Any identified covariates will be controlled for in the subsequent analyses. The same analysis will be used to determine if the intervention group improved on the variables assessed for hypothesis 1 (basic psychological needs and autonomous motivation), 2 (exercise, life satisfaction, participation in daily/social activities, depressive symptoms), and 3 (well-being, and psychosocial predictors of exercise). A regression analysis approach is recommended for analyzing RCT data as this approach can address specific questions arising from the study design, above what could be expected from analysis of variance or other similar types of analyses [68]. As such, two hierarchical multiple regressions will be used to predict changes in participants’ scores between baseline, 6 weeks, and 10 weeks. Specifically, participants’ demographic and injury-related covariates will be entered in the first block, followed by their baseline scores on all study variables in the second block, and finally their group (ie, intervention or control) in the third block. This analysis will be conducted twice: once predicting participants’ scores at 6 weeks, and again predicting their scores at 10 weeks. The differences in variance explained by the model (*r*^2^) and the weight of the regressions (standardized betas) will be used to evaluate whether the model fit improves between Blocks 2 and 3, as well as to evaluate the success of the pilot RCT. In line with the Theoretical Coding Scheme, exploratory analyses will also be conducted to investigate the strength and direction of the relationships between the SDT variables and exercise participation, looking for moderate-to-strong relationships. Correlations of .1, .3, and .5 represent small, moderate, and strong relationships between the variables [69]. If the intervention has a moderate effect on the SDT variables and if the SDT variables have moderate-to-strong relationships with exercise participation, we can then hint at a potential mediation (ie, intervention is having an effect on exercise participation through the SDT variables). Because of the sample size, we do not have sufficient power to run full mediation analyses and it is for this reason it remains exploratory in nature. Descriptive statistics will be conducted for the implementation and feasibility evaluation.

## Discussion

### Principal Considerations

Our results will have higher credibility due to our design. By using an experimental design, specifically an RCT, cause and effect conclusions are more plausible, which are superior to cross-sectional or longitudinal designs. In addition, all our measures have been validated and found to be reliable in past research, increasing the credibility of our findings. We have also clearly outlined our primary, secondary, and tertiary purposes and associated hypotheses, giving transparency to our proposed project. Our specific outcome-based hypotheses are based on theory or past research, which allows for ease of duplicability of the results. The pilot RCT will also aid in identifying any changes that are required to our design, measures, or procedures before running a full-scale RCT. The implementation and feasibility purpose will enable us to determine if any changes to the intervention protocol should be implemented when designing the subsequent full-scale RCT.

Because we are testing a pilot RCT and focusing on the effect size rather than *P* values, we believe that our results will be able to be transferred to a future, larger, interprovincial exercise telerehabilitation intervention. It is only with this pilot RCT that we can reliably estimate the sample size needed for this larger trial. Our intervention components are predetermined as they are based on sound theory (ie, SDT) and from a taxonomy of behavior change and relational techniques [70,71]. We have also used the Theory Coding Scheme to develop our intervention to ensure that it is truly a theory-based intervention.

We have set procedures to control for some biases. For one, the randomization procedure will help to reduce some biases as the control and intervention group should be equivalent. The research assistant will randomize the participants by opening a blinded, prelabeled envelope. Therefore, the research assistant cannot bias group allocation. The second research assistant will also be blinded to the participants’ group allocation when collecting the follow-up data, which should further reduce biases. We are also recruiting from multiple sources, which would allow us to reduce bias that might stem if participants were recruited from only one source and subsequently enhance the generalizability of our results. With the control group having access to the exercise counselor after the study, we hope to reduce the compensatory rivalry bias. We are also avoiding focus on *P* values due to their reliance on sample size [72]. Because of our sample size, if we solely focus on the *P* value we might be making a type II error.

In line with the general limitation of telerehabilitation, our intervention is limited to individuals who are comfortable using a computer and capable of running the Web-based audio-video REACTS software. Given the small sample of this pilot RCT, we will be unable to conduct subsample analyses to investigate whether the intervention had effects for individuals with specific demographic (eg, age, education level) and SCI-related factors (eg, years since injury, adapted home). However, we do plan to control for these variables in our regression models through a correlation matrix. These correlations will enable us to be mindful of these factors when designing the full-scale RCT.

### Knowledge Translation

Results of this study will be translated through three main avenues. First, we plan to share our results with the academic community by presenting the results at SCI, disability, and/or exercise-related scientific meetings, and publishing our results in scientific peer-reviewed journals. Second, we will translate our findings to local adapted exercise centers to inform programs and kinesiologists of our study’s results and approach. Finally, we will share our results with various community organizations who may want to distribute to their larger membership to reach adults with SCI. Our results will therefore be disseminated to academics, the broader SCI community, and relevant professionals.

### Conclusions

This study will be the first to test SDT in an exercise intervention among adults with paraplegia. It is also the first to examine the use of an online video-based telerehabilitation approach in this context. We anticipate that the results of this pilot RCT will support that the intervention is appropriate for adults with SCI and feasible to test in a full-scale RCT.

## Abbreviations

QOL: quality of life
RCT: randomized controlled trial
REACTS: Remote Education, Augmented Communication, Training, and Supervision
SCI: spinal cord injury
SDT: Self-Determination Theory

## Appendix

Multimedia Appendix 1 Comments by the grant reviewers from the Craig H. Neilsen Foundation.
