# Supplementary material for: A Telerehabilitation Approach to Enhance Quality of Life Through Exercise Among Adults With Paraplegia: Study Protocol
Source: JMIR Res Protoc. 2017 Oct 19;6(10):e202. doi: 10.2196/resprot.8047 (PMC5668635; doi:10.2196/resprot.8047)
Supplement: Multimedia Appendix 1 [file resprot_v6i10e202_app1.pdf]

Proposal Information

Applicant: Sweet, Shane
Title Enhancing quality of life through exercise: A tele-rehabilitation appproach
Type/Competition PSR Pilot Grants
Institution McGill University
App # 364137

Committee: PSR 2016 FGA

Summary Statement

Text Admin Summary:
CHNF has conditionally approved funding of this proposal. Please see the reviewer comments for a listing of the strengths and weaknesses identified in your application. The following issue(s) deserve particular attention: The project's use of a video-based tele-rehabilitation platform to implement, motivate and monitor participants across a large community setting, is innovative and will address important gaps in our knowledge between physical activity and psychosocial outcomes. The PI and his team have complementary skills, related research experience, and institutional resources to conduct the study successfully. While the timeline is ambitious, reviewers noted that this project was clearly described and has many strengths and should help identify problems that limit participation, as well as strategies that promote participation. This pilot study will help establish how big an effect the training will have, which is needed to plan any future, larger scale study.

Reviewer Role: Primary (237820)

OVERALL IMPACT
After considering all of the review criteria, summarize the significant strengths and weaknesses of the application. Please state the likelihood that the project scope suits the pilot funding level and will develop an area of work that has the potential to exert a sustained powerful influence on the SCI field and/or be an important contributor to the field of SCI research.

:
The investigators propose to implement a video-based telerehabilitation intervention to promote physical activity among people with SCI, embedded in a self-determination theory framework. The advantages of telerehabilitation to reach the geographically remote regions of Canada is logical. The actual ability to implement this telerehab strategy is not well known at this point and the success of the project hinges in the ability to deploy the telerehab technology. This is a highly ambitious project with numerous individuals involved who will need training and monitoring. The heavy load of interaction and follow-up, although well described, does not seem truly feasible in a one year study. Any incident along the way that sets the project back could be very detrimental to the overall success. This level of ambitiousness reduces enthusiasm for this project.

SIGNIFICANCE
1. Does the project address an important problem or a critical barrier in the field?

2. If the Aims of the project are achieved, how would this work change or enhance current methods, technologies, treatments, services, or interventions?

:
The investigators argue that the relationship between physical activity and quality of life is well substantiated. While this may be true for many individuals, there is a group of people with SCI who find life fulfillment in other arenas that do not involve physical activity - this group does not seem to be addressed in this project and that is a shortcoming. Nonetheless, for those who do desire to engage in physical activity, the project is likely to identify problems that limit participation and could lead to the development of appropriate interventions to facilitate participation. The aims of the project are in alignment with the CHNF mission.

RELEVANCE
The mission of the Craig H. Neilsen Foundation is "to improve the quality of life for those living with spinal cord injury and to support scientific exploration for effective therapies and treatments leading to a cure."

The overall goal for the new Psychosocial Research (PSR) initiative is to lead to "better outcomes for people living with SCI." Emphasis is placed on research directed towards:
a) increased understanding of psychological, social and environmental determinants of health, functioning and activity participation;
b) rehabilitation and habilitation interventions to improve psychological and social functioning, including participation in work, school and other community activities;
c) improved measurements of psychological, social and environmental risk factors, protective factors, processes and outcomes; and
d) identification of critical service gaps, needed data and/or new areas of exploration, within a psychosocial or socioecological context, as defined by, or with input from, people living with SCI.

1. How is this project relevant to the mission of the Foundation?

2. How is this project relevant to the goal of the Psychosocial Research initiative?

:
Alignment with the PSR program and CHNF mission is appropriate for this project. The intent to identify barriers to physical activity and implement a video-based telerehab program is described well, with a great deal of attention given to the theoretical underpinnings supporting this project.

INNOVATION
1. Does the project challenge and seek to shift current research or clinical practice/program intervention paradigms by utilizing novel theoretical concepts,

approaches or methodologies, instrumentation, or interventions?

2. And/or does this application apply concepts, approaches or methodologies, instrumentation, or interventions from another field of research to spinal cord injury?

3. And/or is a refinement, improvement, or new application of theoretical concepts, approaches or methodologies, instrumentation, or interventions proposed?

:
The investigators point out that this project is innovative in that it is using a video-based telerehab platform to implement and monitor physical activity. There is one study by Galea and colleagues that assess video telerehab in the veteran population in the US published in 2006. No other studies specifically addressing this issue were found, so the concept is innovative, although not entirely original. Nonetheless it does address a gap in our knowledge.

INVESTIGATOR(S)
1. Are the PI, collaborators, and other contributors well suited to the project?

2. If the PI is a junior investigator, does he/she have appropriate experience, training and facilities to do the proposed work? If the PI is an established investigator, has he/she demonstrated an ongoing record of accomplishments that have advanced their field(s)? Will this project help establish a junior PI's research program and/or enhance an established program?

:
Dr. Sweet has a clear focus on physical activity and motivation to participate and is well-positioned to lead this project. The support team appears to have the necessary experience with physical activity, behavior and motivation. Dr. Kairy and Ms. Fillion appear to have experience with telerehabilitation techniques.

APPROACH
1. Is the project well-suited to the pilot stage? Are the overall strategy, methodology, and analyses well-reasoned and appropriate to accomplish the Specific Aims of the project?

2. Is the future applicability of the pilot project defined (i.e., does this pilot help lay the groundwork for future studies, define next stages or have the potential to impact current practices and approaches)?

3. Are potential problems, alternative strategies, and benchmarks for success presented? If the project is in the early stages of development, will the strategy establish feasibility and will particularly risky aspects be managed adequately?

4. If the project involves clinical and/or community-based research: 1) are the plans for protection of human subjects from research risks described and adequate; and 2) are the plans for recruitment of patients/participants appropriate?

:
As mentioned previously, this pilot project is very ambitious in terms of the proposed methodology, implementation of the intervention, etc. There is a great deal of work to be done by specific individuals in order to ensure complete data collection and although the investigators have laid out a detailed timeline, there are concerns that not all activities can be accomplished in the time frame of funding. Particularly in weeks 6 through 12 in the second quarter and weeks 1-2 in the third quarter, the work load of the activity counselor is quite daunting. Should this pilot be successful, however, a full scale RCT could be proposed. The investigators have identified these problem areas as well as others and have offered reasonable approaches to address issues as they come along, such as building in extra time for data collection if needed. I did not see a plan for protection of human subjects.

ENVIRONMENT
1. Will the institutional environment in which the work will be done contribute to the probability of success?

2. Are the institutional support, physical equipment and other resources available to the investigators adequate for the project proposed?

3. Will the project benefit from unique features of their environment, subject populations, or collaborative arrangements?

:
There appears to be appropriate and adequate institutional support to complete this project. There is substantial salary support provided for some of the staff from their respective institutions.

NON-SCORED CRITERIA
(PLEASE NOTE THAT THE SCORE FOR THIS NON-SCORED SECTION MUST BE A "50 - NON-SCORED." PLEASE DISREGARD SCORING OPTIONS 1-9 FOR THIS SECTION ONLY.)

Please provide any important or relevant comments on each of the 4 non-scored criterion below.

1. Budget

2. Ethics/Safety

3. Other

4. Additional Comments for the Applicant

5. RESUBMISSION: When reviewing a Resubmission, the committee will evaluate the application as now presented, taking into consideration the responses to comments from the previous scientific review group and changes made to the project.

:
Statement of ethics/safety is missing - no mention of subject protections.

Reviewer Role: Secondary (138888)

OVERALL IMPACT
After considering all of the review criteria, summarize the significant strengths and weaknesses of the application. Please state the likelihood that the project scope suits the pilot funding level and will develop an area of work that has the potential to exert a sustained powerful influence on the SCI field and/or be an important contributor to the field of SCI research.

:
Many strengths--they document the need, the connection between PA and psychosocial outcomes, the current state of the science and propose a study that fills a next doable step that fits within this funding mechanism. They propose a small RCT that is clearly tied to the existing evidence base and extends this with relevant theory. They provide all the needed details regarding design, recruitment, attrition, rationale for the control group type, etc. They are highly experienced and have already done innovative work in this area. They have a team of scientists with complementary skills and trainees to help run the study. Weaknesses: no significant weaknesses.

SIGNIFICANCE
1. Does the project address an important problem or a critical barrier in the field?

2. If the Aims of the project are achieved, how would this work change or enhance current methods, technologies, treatments, services, or interventions?

:
The authors do a nice job of demonstrating an empirical connection between physical activity (PA) and various aspects of QOL. They also show that PA is low in people with SCI. They show that a telerehab approach should be used to overcome barriers to PA interventions.

RELEVANCE
The mission of the Craig H. Neilsen Foundation is "to improve the quality of life for those living with spinal cord injury and to support scientific exploration for effective therapies and treatments leading to a cure."

The overall goal for the new Psychosocial Research (PSR) initiative is to lead to "better outcomes for people living with SCI." Emphasis is placed on research directed towards:
a) increased understanding of psychological, social and environmental determinants of health, functioning and activity participation;
b) rehabilitation and habilitation interventions to improve psychological and social functioning, including participation in work, school and other community activities;
c) improved measurements of psychological, social and environmental risk factors, protective factors, processes and outcomes; and
d) identification of critical service gaps, needed data and/or new areas of exploration, within a psychosocial or socioecological context, as defined by, or with input from, people living with SCI.

1. How is this project relevant to the mission of the Foundation?

2. How is this project relevant to the goal of the Psychosocial Research initiative?

:
The investigators do an excellent job of connecting the empirical dots between physical activity and the sorts of outcomes this funding mechanism aims at. They identify critical gaps in the literature and their study is precisely focused on a next doable step that will move the science forward and fit into this funding (one year) niche.

INNOVATION
1. Does the project challenge and seek to shift current research or clinical practice/program intervention paradigms by utilizing novel theoretical concepts, approaches or methodologies, instrumentation, or interventions?

2. And/or does this application apply concepts, approaches or methodologies, instrumentation, or interventions from another field of research to spinal cord injury?

3. And/or is a refinement, improvement, or new application of theoretical concepts, approaches or methodologies, instrumentation, or interventions proposed?

:
Just the right degree of innovation--theory and evidence based next step. Does not open up a whole new area, but moves forward in one we know about but in which there is more work to do.

INVESTIGATOR(S)
1. Are the PI, collaborators, and other contributors well suited to the project?

2. If the PI is a junior investigator, does he/she have appropriate experience, training and facilities to do the proposed work? If the PI is an established investigator, has he/she demonstrated an ongoing record of accomplishments that have advanced their field(s)? Will this project help establish a junior PI's research program and/or enhance an established program?

:
This group demonstrates that they have exactly the sort of experience and infrastructure and pilot data with which to conduct this study. Their prior research demonstrates that they have the resources to carry out a study like this.

APPROACH
1. Is the project well-suited to the pilot stage? Are the overall strategy, methodology, and analyses well-reasoned and appropriate to accomplish the Specific Aims of the project?

2. Is the future applicability of the pilot project defined (i.e., does this pilot help lay the groundwork for future studies, define next stages or have the potential to impact current practices and approaches)?

3. Are potential problems, alternative strategies, and benchmarks for success presented? If the project is in the early stages of development, will the strategy establish feasibility and will particularly risky aspects be managed adequately?

4. If the project involves clinical and/or community-based research: 1) are the plans for protection of human subjects from research risks described and adequate; and 2) are the plans for recruitment of patients/participants appropriate?

:
They take a logical step-wise approach to science. Having done an open trial, now they want to do a small RCT. They use a theory-based approach to build the intervention. They have pilot data from a previous open trial. Their plan is to pilot the intervention in preparation for a fully powered efficacy trial. Very thoughtful systematic approach to scientific progress. They take extra unprecedented steps to ensure their approach is theory based.

ENVIRONMENT
1. Will the institutional environment in which the work will be done contribute to the probability of success?

2. Are the institutional support, physical equipment and other resources available to the investigators adequate for the project proposed?

3. Will the project benefit from unique features of their environment, subject populations, or collaborative arrangements?

:
Seems good. Good subject availability. Post-docs available to help run study. Lab space available. Prior work indicates resources are there.

NON-SCORED CRITERIA
(PLEASE NOTE THAT THE SCORE FOR THIS NON-SCORED SECTION MUST BE A "50 - NON-SCORED." PLEASE DISREGARD SCORING OPTIONS 1-9 FOR THIS SECTION ONLY.)

Please provide any important or relevant comments on each of the 4 non-scored criterion below.

1. Budget

2. Ethics/Safety

3. Other

4. Additional Comments for the Applicant

5. RESUBMISSION: When reviewing a Resubmission, the committee will evaluate the application as now presented, taking into consideration the responses to comments from the previous scientific review group and changes made to the project.

:
No dedicated faculty time from what I can see. Not sure what their commitment is

©2007-2017 Altum, Inc. All rights reserved	Contact Us	Terms of Service	Acceptable Use Policy	Privacy Policy
